# Availability, functionality and access of blood pressure machines at the points of care in public primary care facilities in Tororo district, Uganda

**DOI:** 10.4102/safp.v63i1.5118

**Published:** 2021-01-11

**Authors:** Innocent K. Besigye, Vicent Okuuny, Mari Armstrong-Hough, Anne R. Katahoire, Nelson K. Sewankambo, Robert Mash, Achilles Katamba

**Affiliations:** 1 Department of Family Medicine, Makerere University, Kampala, Uganda; 2 School of Global Public Health, New York University, New York, United States of America; 3 Department of Child Health and Development Centre, Makerere University, Kampala, Uganda; 4 Department of Medicine, Makerere University, Kampala, Uganda; 5 Department of Family and Emergency Medicine, Stellenbosch University, Cape Town, South Africa

**Keywords:** hypertension, primary care, primary healthcare, health facilities, blood pressure machine

## Abstract

**Background:**

Early diagnosis of hypertension prevents a significant number of complications and premature deaths. In resource-variable settings, diagnosis may be limited by inadequate access to blood pressure (BP) machines. We sought to understand the availability, functionality and access of BP machines at the points of care within primary care facilities in Tororo district, Uganda.

**Methods:**

This was an explanatory sequential mixed-methods study combining a structured facility checklist and key informant interviews with primary care providers. The checklist was used to collect data on availability and functionality of BP machines within their organisational arrangements. Key informant interviews explored health providers’ access to BP machines.

**Results:**

The majority of health facilities reported at least one working BP machine. However, Health providers described limited access to machines because they are not located at each point of care. Health providers reported borrowing amongst themselves within their respective units or from other units within the facility. Some health providers purchase and bring their own BP machines to the health facilities or attempted to restore the functionality of broken ones. They are motivated to search the clinic for BP machines for some patients but not others based on their perception of the patient’s risk for hypertension.

**Conclusion:**

Access to BP machines at the point of care was limited. This makes hypertension screening selective based on health providers’ perception of the patients’ risk for hypertension. Training in proper BP machine use and regular maintenance will minimise frequent breakdowns.

## Background

Cardiovascular diseases (CVDs) are amongst the most common non-communicable diseases (NCDs) and are on the rise in sub-Saharan Africa.^1,2^ They account for one-third of global deaths, with nearly 85% of the CVD mortality occurring in low- and middle-income countries (LMICs).^3^ Hypertension is the leading cause of CVD morbidity and mortality. Globally, 54% of strokes and 47% of coronary heart diseases are related to hypertension.^4^

Hypertension is the strongest modifiable risk factor for NCDs: if diagnosed and properly managed early, a significant number of complications and premature deaths can be prevented.^5^ Unfortunately, most people living in LMICs are unaware of their hypertension status. Those who are diagnosed are more likely to be poorly controlled and face higher incidence of complications and deaths than their counterparts in high-income countries.^6^ In Uganda, the prevalence of prehypertension and hypertension is 36.9% and 26.4%, respectively. Yet, only 7.7% of those with hypertension in Uganda are aware of their status.^7^ Focusing on early diagnosis in primary care settings, where most patients are seen and cared for, can greatly improve awareness and avert complications and early mortality in high-burden, low-awareness LMIC settings like Uganda.

The World Health Organization (WHO) recommends blood pressure (BP) measurement to increase awareness and improve health outcomes in the management of hypertension in resource-limited settings.^8^ However, the scarcity of basic equipment, such as BP measuring machines, may limit the implementation of this recommendation. Health providers must cope with this scarcity to optimally screen for hypertension.^9,10^ This study aimed to establish the availability and functionality of BP machines in primary care facilities and explore their access by health providers at the points of care serving a high hypertension prevalence population in Tororo district of Eastern Uganda.

## Methods

### Study design

To understand the availability of BP machines in primary care facilities and the ability of health providers to access them at the point of care, we designed an explanatory, sequential, mixed-methods study using a structured checklist and key informant interviews with health providers. The tools for data collection were designed using the Capability, Opportunity and Motivation, Behaviour (COM-B) model.^11^

### Study setting

The study was conducted in public primary care facilities in Tororo district in Eastern Uganda. Primary care facilities have multiple points of care within their organisational arrangements providing different services, including in-patient services, general outpatient services, antenatal care and disease-specific services, such human immunodeficiency virus (HIV) and tuberculosis, amongst others.

The burden of hypertension in Eastern Uganda is the second highest in the country (26.4%) and is similar to that of the highest burden region, Central Uganda (28.5%). The Ugandan primary care system is organised hierarchically into general hospitals (GHs), health centre 4s (HC4), health centre 3s (HC3), health centre 2 dispensaries (HC2) and village health teams (VHTs). Tororo district has one GH, 4 HC4s, 18 HC3s and 31 HC2s. These facilities form the district health system (DHS). In Uganda, primary care is provided throughout the whole DHS, from the VHT up through the GH. Primary care providers include nurses, clinical officers and non-specialist doctors referred to as medical officers.

### Participant selection

This study included primary care providers assigned to the outpatient department of the GH, HC4 facilities or HC3 facilities. Health centre II dispensaries were excluded because they lack the capacity to provide extensive clinical assessment of patients. Research assistants identified and purposively selected primary care providers to include those working in the different levels of health facilities. The providers were contacted by telephone and invited to participate in the study. All the providers contacted agreed to participate in the study after understanding its purpose. An appointment was set and the research assistants travelled to the respective workplaces of the participants to carry out the interviews between November and December 2018.

### Data collection

An interviewer-administered checklist was used to collect data on the availability and functionality of BP machines in each facility. A semi-structured interview guide was used to carry out the interviews. The interview guide probed participants regarding how hypertension patients are identified at the facilities; how health providers screen, diagnose and manage patients; and what problems are experienced in carrying out these procedures at the facility. Interviews were conducted in English and audio-recorded using a digital voice recorder. The interviews were conducted by two trained research assistants: a newly qualified family physician (male) familiar with primary care and a nurse (female) with prior experience working in a GH. Data saturation was deemed to have been achieved when no new information was being generated from the last three interviews. Data collection was stopped upon reaching saturation.

### Data analysis

Data from the checklist were entered into Epidata software program and summarised using percentages. Interviews were transcribed verbatim. The transcripts were imported into Atlas ti version 8.5 and their content was analysed using an inductive approach. The principal investigator (PI) familiarised himself with the transcripts through repeated reading, making notes and memos. The PI applied open coding, after which the code tree and code applications were reviewed and revised with colleagues. New codes were then applied to the rest of the data. Subsequently, the codes were grouped into categories and themes were identified. After content analysis was completed, the PI chose illustrative quotations for each theme.

### Ethical consideration

This study was approved by Makerere University School of Medicine Research and Ethics Committee (#REC REF 2017-170) and the Uganda National Council for Science and Technology. The Tororo District Health Office granted administrative clearance to carry out the study. All participants provided written informed consent to participate in the study.

## Results

Eighteen health facilities were surveyed. One facility reported having no BP machine at all, whilst the remaining 17 (94.0%) facilities reported having at least one working BP machine. One (6.0%) health facility reported annual calibration of the BP machines; the remainder (94%) reported no regular servicing of machines. Thirteen (76.0%) facilities used digital machines. Ten (55.6%) health facilities had at least one BP machine not working on the day they were surveyed (see Table 1).

Sixteen primary care providers participated in key informant interviews (Table 2). The participants were mostly women (*N* = 11; 68.8%) working in HC3 (*N* = 7; 43.8%) or GH (*N* = 6; 37.5%). Participating providers reported an average of 14 years of work experience in the district. Participants included nurses qualified with certificates and diplomas (*N* = 11; 68.8%), clinical officers with diploma (*N* = 4; 25.0%) and medical officers with a degree (*N* = 1; 6.2%).

Although there was at least one functional BP machine in almost all health facilities surveyed, health providers said that their access to BP machines remained limited. Whilst available at the facility, the number of BP machines was insufficient. Therefore, a machine could not be located at each point of care:

‘The BP machines are not enough. We don’t have enough (BP machines) to cover all those areas where there are entry points for patients.’ (KI01, GH, female nurse, 12 years in service)

‘We have only one for the whole facility, maternity and OPD, HIV clinic.’ (KI09, HC3, female nurse, 11 years in service)

The providers perceived that frequent breakdown, together with the failure of the maintenance team to regularly repair them, contributed to this limited access to BP machines:

‘Maintenance of those equipment is done but it takes a long time. At times, it can take about 3 months, 4 months and that period is long. Yes, I wish they could do it regularly especially for the BP machines.’ (KI03, GH, male clinical officer, 19 years in service)

The participants perceived the maintenance team, which is based in the study region, to be uncommitted. Providers reported that the maintenance team do not come regularly to repair the broken machines, even when they are called upon:

‘Yeah, they [service team] are contacted and come but at times they delay to come.’ (KI10, HC3, female nurse, 14 years in service)

More frequent breakdowns were attributed to inappropriate use of these machines by health providers because of lack of skills or training. These breakdowns are quite common with machines that use mercury. Mercury must be put back into the tank after use and before folding the machine in order to avoid breaking in the column, but some health workers are unfamiliar with these usage guidelines:

‘Then, sometimes we have BP machines that we don’t know how to use like the other old mercury machines. People do not know how to put the mercury back.’ (KI01, GH, female nurse, 12 years in service)

Health providers explained that because of the difficulties in using the manual mercury BP machines, they would prefer the digital ones:

‘These days people are moving away from these manual ones and they opt for the digital ones which are more easier to use.’ (KI02, GH, female nurse, 11 years in service)

Because BP machines were not readily available at the point of care, health providers resorted to improvisation to measure patients’ BPs. Firstly, health providers described moving around the facility looking for BP machines to measure a patient’s BP:

‘We walk around the facility, we just walk and go to where you think there is a BP machine and you get the patient’s pressure taken.’ (KI03, GH, male clinical officer, 19 years in service)

Therefore, health providers resort to borrowing amongst themselves within their respective units or from other units within the facility:

‘We then borrow from other wards or areas that have BP machines.’ (KI01, GH, female nurse, 12 years in service)

However, providers complained that this borrowing is time-consuming and sometimes causes discomfort to the borrower and the ones being borrowed from. Therefore, this borrowing phenomenon is a bad experience to the providers:

‘Ah its nasty, why, it’s time-consuming probably you send a nurse there and they say we are also using it, you even reach there and they tell you the cells are off.’ (KI14, GH, male clinical officer, 11 years in service)

Moreover, sometimes, the borrowing is unsuccessful because the borrower finds the machine being used or not functional:

‘The people with the BP machines are not comfortable. Actually, in the line there could be another person waiting; if you go with a BP machine, already someone comes and begins borrowing.’ (KI03, GH, male clinical officer, 19 years in service)

Providers also explained that the presence of students from various health professional training institutions, particularly the nursing schools, facilitates the borrowing process. They described sending student nurses around the facility to look for machines:

‘We send students to look for BP machines around the facility. When students are not around, it is difficult to find someone around to go and borrow from other units as everyone is busy with their work.’ (KI12, GH, male medical officer, 13 years in service)

The presence of students in the facilities is also helpful because it is an institutional requirement for students to buy and own a BP machine. Therefore, students become a resource for the providers. However, students are not in these facilities all the time. When the students are absent, it may become much more difficult for a provider to locate a working BP machine:

‘It is a requirement that when they (students) report for hospital, they come with BP machines and we teach them how to use them. We use their BP machines to monitor patients. The only challenge, when the students are not there. That’s when we have problems.’ (KI01, GH, female nurse, 12 years in service)

Finally, some health providers reported using their own money to purchase BP machines for use in public health facilities:

‘At times, there is when we carry our own from home.’ (KI10, HC3, female, 14 years in service)

‘… Some of us have to buy and carry our own because we need to care for this patient, we need to monitor this patient because you will not give this patient medication without monitoring the levels of the blood pressure.’ (KI14, GH, male clinical officer, 11 years in service)

Providers also described attempts to restore the functionality of broken BP machines because working machines were unavailable to them. However, health providers pointed out that they lack the skills necessary to repair broken machines and said that they seek help from around the town:

‘But at times, what we can do, at times it’s the piece of cloth. At times it can be the covering, we take them in town here. I have taken several times. In town, then the man works on it because at times, it [is] just the piece of cloth that is torn, so we just take and then it is covered well and then we keep using as we wait for the maintenance group, but if it is a major part like this one which uses mercury and you find the metals are broken, it becomes a problem, because we have to wait for the maintenance department to come.’ (KI08, HC4, female nurse, 25 years in service)

Because of the difficulty and frustration involved in locating a BP machine, providers said that they did not regularly check BP for all patients. Rather, they focused primarily on measuring BP for those patients whom they already suspected were hypertensive. Health providers said that they were compelled to look for BP machines when they perceived a patient to be at risk of hypertension because of obesity, age or comorbidity:

‘You can always borrow when you have somebody you suspect to be having hypertension. You can go and borrow from another unit but you know what it takes.’ (KI02, GH, female clinical officer, 11 years in service)

‘Yes, because it is difficult to take pressure for everyone at the facility. There are some people whom we think are prone like someone is obese, someone is pregnant, we try to take the pressure of these people.’ (KI03, GH, male clinical officer, 19 years in service)

Therefore, providers explained that not all patients are screened for hypertension.

## Discussion

The World Health Organization recommends measurement of BP for every patient, particularly in resource-variable settings.^8^ This is not possible unless BP machines are available at all points of care. In our study, we found that BP machines are generally present within health facilities but not accessible to health providers at the point of care. To access the BP machines that are available within their facility, providers reported having to invest time and effort to locate, borrow and return functional machines. This makes it difficult for health providers to screen every patient for hypertension. Our findings demonstrate that facility measures of availability may be misleading and not a reliable indicator of health providers’ access to these machines. Blood pressure machine shortage is a key contributor to the hypertension-relevant equipment shortage that has been documented in other studies in low-resource settings.^10,12^ This shortage, in turn, contributes to the low levels of awareness and low proportion of patients screened for hypertension within health facilities in settings like Uganda.

As a result of limited access to BP machines at points of care and the effort required to locate working machines elsewhere, health providers in our study reported selectively screening patients for hypertension based on their perceptions of the patient’s risk. If providers screen only patients who are elderly, obese or known to have conditions like diabetes mellitus, many patients with hypertension are likely to be missed. Hypertension is a painless and often a symptomless disease condition. Prevalence surveys also suggest that it is alarmingly common in sub-Saharan Africa, even amongst those who providers may not presume to be at the highest risk.^3,7,13^ These missed hypertension patients are likely to present with complications later in their lives.

Our findings also suggest that frequent machine breakdown coupled with unresponsive equipment maintenance services contributes to the scarcity of BP machines. Frequent breakdowns increase the costs of running health facilities. Buying and maintaining equipment is one of the largest expenses for health facilities. A committed team that regularly services and maintains equipment could minimise expenses and improve patient care. In our study, some health providers reported seeking the services of local tailors, an attempt that is inefficient and costly to the providers and also not sustainable. Regular maintenance and improved training of health providers regarding proper storage of medical equipment could reduce the cost and improve sustainability.

Finally, health providers’ preference for digital BP machines and their increased availability should be of concern. Studies have shown that digital BP machines are less accurate compared to standard mercury devices.^14^ Digital BP machines may underestimate BP, resulting in misclassification of hypertension.^15,16^ Many patients are likely to be misdiagnosed as non-hypertensive, with a significant risk to complications. Moreover, digital devices require recurrent expenditures for batteries or cells, an important consideration in resource-variable settings. On the other hand, increased use of digital BP machines reduces the incidence of white coat hypertension. Therefore, it will not be wise to discourage their use altogether.

According to the Primary Health Care Performance Initiative (PHCPI), the availability of equipment and supplies is a key input into quality primary care.^17^ Blood pressure measuring devices in most primary care systems are considered to be a basic and essential piece of equipment that should be available in every consulting room. The non-availability of basic equipment in the Ugandan primary care system is a sign that the system as a whole may be weak and may have other essential supply and equipment deficiencies – BP machines could be seen as a sentinel item.

## Strengths and limitations of the study

This study had several strengths. The study covered all major levels of primary care and involved almost all health facilities in a single district and included all cadres of primary care providers. Its mixed-methods design integrated facility-level observations of availability with provider-level perspectives on access. This approach allowed us to characterise a paradox of access to an essential diagnostic device: BP machines are accurately described as ‘available’ in facility-level surveys whilst simultaneously remaining inaccessible to most providers when they need them most.

This study has some limitations also. We did not assess the proportion of patients screened for hypertension to establish the magnitude of non-screening in primary care facilities. It was also not possible to establish which points of care have most regular access to BP machines because they are only available at facility level and shared between units.

## Recommendations

Blood pressure machines must be accessible at the point of care to support timely diagnosis and management of hypertension. They should be regularly inspected, calibrated and repaired. To minimise frequent breakdowns, health providers should be trained in the proper use of BP machines. Future research should seek to quantify the possible contribution of BP machines’ limited access to high numbers of undiagnosed hypertension patients and low hypertension status awareness.

## Conclusion

Public primary care facilities in Tororo district have insufficient numbers of functional BP machines to provide quality primary care for hypertension patients. Most consulting rooms do not have a BP machine and this undermines clinical care of at-risk patients as well as the ability to screen for hypertension. This made screening for hypertension sub-optimal and selective based on health providers’ perceptions of the patients’ risk for hypertension.

## Figures and Tables

**TABLE 1:** Availability and functionality of blood pressure machines within health facilities.

Variable	Number of health facilities	Percentage of health facilities
**Presence of BP machine**		
Yes	17	94.4
No	1	5.6
**Servicing or calibration**		
Yes/once a year	1	5.9
No	16	94.1
**BP machine type**		
**Number of aneroid**		
2	1	11.1
0	16	88.9
**Number of digital**		
0	4	23.5
1	3	17.6
2	7	41.2
3	2	11.8
4	1	5.9
**Number of mercury**		
0	8	47.0
1	7	41.2
3	2	11.8
**Working status**		
**Number of BP machines working**		
0	1	5.9
1	8	47.0
2	6	35.3
3	1	5.9
5	1	5.9
**Number of BP machines not working**		
0	7	41.2
1	6	35.3
2	4	23.5

BP, blood pressure.

**TABLE 2:** Participants details of key informant interviews.

No.	Facility level	Sex	Age (years)	Cadre	Highest qualification	Years in district service
KI01	GH	Female	55	Nursing Officer	Diploma	12
KI02	GH	Female	42	Clinical Officer	Diploma	11
KI03	GH	Male	52	Clinical Officer	Diploma	19
KI04	HC3	Female	37	Enrolled Nurse	Certificate	14
KI05	HC3	Female	38	Nursing Officer	Diploma	11
KI06	HC3	Female	28	Enrolled Nurse	Certificate	6
KI07	GH	Female	46	Nursing Officer	Diploma	15
KI08	HC4	Female	48	Nursing Officer	Diploma	25
KI09	HC3	Female	39	Enrolled Nurse	Certificate	11
KI10	HC3	Female	37	Enrolled Nurse	Certificate	14
KI11	HC4	Female	27	Clinical Officer	Diploma	5
KI12	GH	Male	45	Medical Officer	Degree	13
KI13	GH	Female	50	Nursing Officer	Diploma	24
KI14	GH	Male	41	Clinical Officer	Diploma	11
KI15	HC3	Female	38	Enrolled Nurse	Certificate	15
KI16	HC3	Male	36	Enrolled Nurse	Certificate	12

GH, general hospitals; HC, health centre.
